# A curious case of cough: Mounier-Kuhn syndrome in a Namibian female patient

**DOI:** 10.11604/pamj.2020.36.56.23375

**Published:** 2020-06-02

**Authors:** Mercy Juliette Mkandawire, Nobert Makombe Muramira, Ngalawi Mraba

**Affiliations:** 1 Department of Internal Medicine, Ongwediva Medipark Teaching Hospital, Ongwediva, Namibia,; 2 Faculty of Health Sciences, Department of Medicine, University of Namibia, Windhoek, Namibia

**Keywords:** Mounier-Kuhn syndrome, HIV, airway dilatation, Namibian female, tracheomalacia

## Abstract

We report a case of a 43-year-old female who presented with a history of recurrent productive cough, since her teenage years. Her associated symptoms included dyspnoea, occasional pleuritic chest pain and rarely, constitutional symptoms. Treated numerous times for lower respiratory tract infections, her symptoms would improve after antimicrobial therapy, but always recurred. She had a background of HIV infection and was virologically suppressed on antiretroviral therapy for nine years. Investigations revealed an active pseudomonas infection and high-resolution computed tomography scan (HRCT) and bronchoscopy confirmed features of Mounier-Kuhn syndrome. The patient was treated accordingly with positive airway pressure, mucolytic agents and chest physiotherapy aimed at aiding mucus clearance and received pneumococcal and influenza vaccines. Mounier-Kuhn syndrome, though rare, should be considered in the differential diagnosis of patients with recurrent lower respiratory tract infections. In Africa, more cases may be identified and treated appropriately with timely investigation and treatment.

## Introduction

Mounier-Kuhn syndrome or congenital tracheobronchomegaly, is a rare airway disease of uncertain etiology characterised by abnormal dilatation of the trachea and main bronchi. The clinical condition is associated with recurrent respiratory tract infections and symptoms similar to those of asthma and chronic pulmonary airway disease [1]. A clinical entity comprising of dilated airways was first described in 1897 by Czyhlarz [2], a pathologist, who described autopsy findings. Mounier-Kuhn, in 1932, then elaborated on the findings, relating these to specific endoscopic and radiographic findings and associated recurrent respiratory infections [3]; hence the coining of the Mournier-Kuhn syndrome (MKS). Approximately 400 cases of MKS have been reported worldwide, with no specific epidemiologic studies [1]. It has been thought that MKS has a male preponderance with 8:1 male to female ratio [4]. The fact that our patient is female and has HIV infection as a comorbidity, makes this an even rarer scenario, with a limited number of such cases reported [5]. To our knowledge, no cases have been reported in sub-Saharan Africa.

## Patient and observation

A female teacher, 43 years of age, presented with a history of recurrent productive cough, which she has had since her teenage years. Her associated symptoms included dyspnoea, occasional pleuritic chest pain and rarely, constitutional symptoms. She had been treated numerous times for lower respiratory tract infections, including bronchitis and pneumonia and had received empiric tuberculosis treatment on two occasions. Her symptoms would improve after antimicrobial therapy, but always recurred. She had a background of HIV infection and was virologically suppressed on antiretroviral therapy for nine years. Her drug regimen included tenofavir/emtricitabine/efavirenz and no other chronic medications. She reported no known allergies. She was a non-smoker and had no history of illicit drug usage. No significant surgical history or relevant family medical history was ascertained. On examination, she was not in respiratory distress and had few sparse bibasal wheezes. Her laboratory workup showed a white cell count of 3.1x10^9^/L (4.0-11.0), haemoglobin of 12.3g/dl (12.0-16.0) and platelets of 173x10^9^/L (140-420), C-reactive protein (CRP) was 27mg/L (< 5.0) and erythrocyte sedimentation rate (ESR) was normal. Renal and liver functions were biochemically normal, and CD4 count was 728cells/mm^3^ (700-1100) with HIV viral load <40 copies/ml.

**Figure 1:**
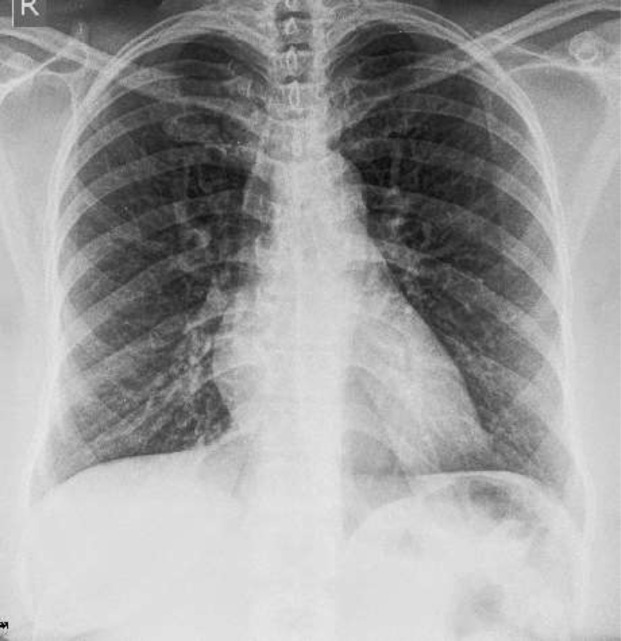
chest X-ray showing bilateral lower zone infiltrates

**Figure 2:**
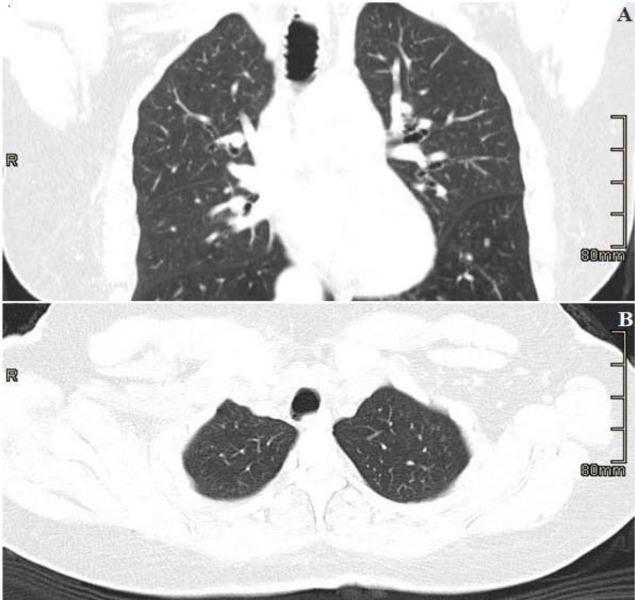
A) HRCT scan images showing prominent trachea; B) posterior diverticuli

**Figure 3:**
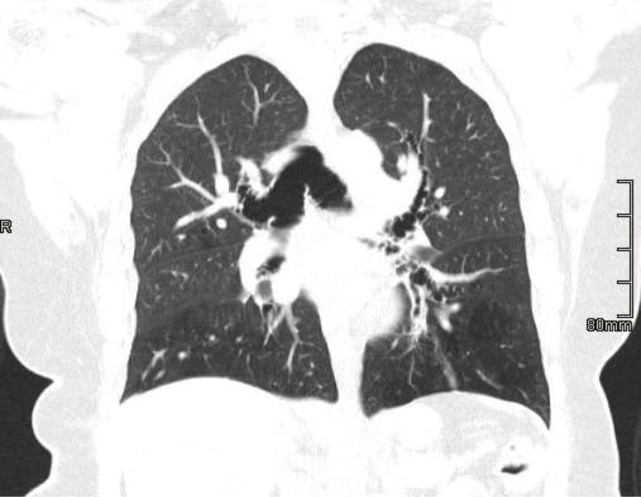
HRCT scan image highlighting bronchial dilatation (right measuring 22.5mm, left 20.4mm), varicose bronchiectasis, centrilobar, emphysema and basal fibrosis

**Figure 4:**
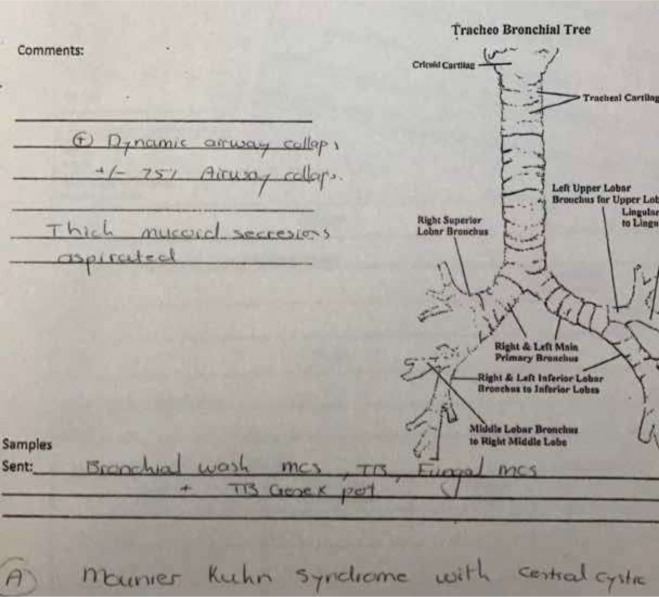
bronchoscopy report confirming MKS: courtesy of Dr. W. Bruwer

Sputum microscopy and culture confirmed heavy growth of sensitive pseudomonas species. Radiological investigation with chest X-ray, posterior-anterior view, showed increased bronchovascular markings and features of an active infection (Figure 1). Owing to the recurrent nature of her symptoms, a high-resolution computed tomography scan (HRCT) was done and revealed the following: prominent trachea with posterior diverticuli (Figure 2); as well as bronchial dilatation, with diameters of right and left measuring 22.5mm and 20.4mm, respectively. Also reported were varicose bronchiectasis, centrilobar emphysema and basal fibrosis (Figure 3). Pulmonary function test (pre-bronchodilator spirometry) was done and reported a moderate - severe restrictive pattern; FVC = 1.86L, FEV1 = 1.55L, FEV1/FVC = 85.5%. After treatment of the acute infection, a bronchoscopy was performed, confirming a dilated trachea and bronchial tree and further described tracheomalacia (collapse of trachea and bronchi during expiration) and the presence of thick mucoid secretions and plugs (Figure 4). Bronchoalveolar lavage samples were collected and analysis revealed no bacterial growth and negative mycobacterium tuberculosis on GeneXpert^®^ and culture.

## Discussion

We report a case of a 43 years old, Namibian female, with Mounier-Kuhn syndrome and a background of HIV infection. The aetiology of MKS is generally unclear, although genetic predisposition to congenital tracheobronchomegaly, as well as environmental factors are thought to play a role. Familial recessive inheritance has been reported [4], but possible secondary causes or associations with various connective tissue disease including Ehlers-Danlos syndrome, Marfan´s syndrome, ataxia-telangiectasia, Bruton-type agammaglobulinemia, ankylosing spondylitis, cutis laxa and light chain deposition disease have also been described [6]. This patient had no relatable family history. She had a negative screen for connective tissue diseases and a normal autoimmune profile. The diagnosis of MKS can be based on clinical as well as chest X-ray and CT scan findings (gold standard). The airway measurements highlighted in Table 1 are diagnostic [7, 8]. CT scan findings are varied and help to provide a staging system for MKS. The radiological society has described three stages of MKS as follows [7, 9]: Type 1: minimal symmetrical dilatation; Type 2: pronounced tracheal dilatation and diverticula; Type 3: marked tracheal and bronchial dilatation and diverticular extending until distal bronchi bilaterally.

**Table 1:** MKS diagnostic criteria

Structure	Transverse Diameter (mm) Diagnostic criteria	Patients Measurements (mm)
Trachea	>30	20.2
Right bronchus	>20	22.5
Left bronchus	>18	20.5

The diagnosis of such a rare condition can be challenging in our setting, due to multiple factors. Limited access to CT imaging can be considered an important factor in the delay of diagnosis. The north of Namibia, which hosts approximately 60% of the population, currently has three CT scan machines available; one in the state sector and two in the private sector, of which our hospital, Ongwediva Medipark, hosts one. Also, limited access to pulmonology services - such as bronchoscopy, which aid in confirming this diagnosis again - contributes to this delay. During the time of investigating this case, the whole of Namibia had one pulmonologist based in the capital city of Windhoek. We entertained a range of differential diagnoses during workup including chronic obstructive airways disease, bronchiectasis, pulmonary tuberculosis, connective tissue disorders; some of which were ruled out with investigations described and did not explain the case findings in their entirety. Another factor is that, in the setting of HIV infection, one may be more inclined towards investigating and diagnosing the commonly seen respiratory infections and disregarding a more thorough investigative approach.

The pulmonary manifestations of HIV infection have been well described in literature. Cases reported so far of MKS in individuals with HIV infection seem to have other possible confounding risk factors such as tobacco and recreational drug use, as noted by Fletcher *et al*. [5]. Another case report described MKS in a patient that had profound immunosuppression with a CD4 count of 3cells/mm^3^ [10]. These additional factors were not present in our case and this could perhaps account for the comparatively mild type of MKS seen. Krustins *et al*. in their review, listed HIV infection as one of the conditions that is most likely to be a separate entity if occurring concurrently with MKS [1]. Other conditions listed in this category include malignancies, adrenal insufficiency and end stage renal failure among others. Our patient was virologically suppressed with a good CD4 cell count, therefore, it seems unlikely that underlying HIV infection would be the sole cause of her recurrent infections. Other associations between MKS and HIV have yet to be conclusively described and further study may be required in this regard. MKS can cause significant morbidity and negatively impact the quality of life of a patient.

Diagnostic delays may lead to a patient developing more severe disease, with irreversible lung fibrosis and worsening respiratory failure [5, 11]. Once the diagnosis is established, specific measures that have been proven to reduce progression and morbidity can be offered in a timely manner. These include; effective treatment of infections, positive airway pressure, use of mucolytic agents and chest physiotherapy aimed at aiding mucus clearance. Pneumococcal and influenza vaccines are recommended for these patients [1, 11, 12]. The usefulness of prophylactic antibiotics in this regard is yet to be determined. Surgical options, such as airway stenting and tracheobronchoplasty, have proven to be useful in some more severe cases [13]. Our patient benefitted from positive airway pressure management and adjuvant therapy as described above and did not require any surgical interventions. A year after her diagnosis and appropriate management, she objectively reports fewer respiratory tract infections. She follows up with the pulmonologist every 6 months.

## Conclusion

MKS, though rare, should be considered in the differential diagnosis of patients with recurrent lower respiratory tract infections. In Africa, more cases may be identified and treated appropriately with timely investigation and treatment.
